# CYP2B6*6, CYP2B6*18, Body weight and sex are predictors of efavirenz pharmacokinetics and treatment response: population pharmacokinetic modeling in an HIV/AIDS and TB cohort in Zimbabwe

**DOI:** 10.1186/s40360-015-0004-2

**Published:** 2015-03-27

**Authors:** Milcah Dhoro, Simbarashe Zvada, Bernard Ngara, Charles Nhachi, Gerald Kadzirange, Prosper Chonzi, Collen Masimirembwa

**Affiliations:** Department of Molecular Sciences, African Institute of Biomedical Science and Technology, Dominion House, 211 Herbert Chitepo Street, P.O. Box 2294, Harare, Zimbabwe; Department of Clinical Pharmacology, College of Health Sciences, University of Zimbabwe, Harare, Zimbabwe; Department of Medicine, Division of Clinical Pharmacology, Faculty of Medicine and Health Sciences, Stellenbosch University, Stellenbosch, South Africa; Department of Medicine, College of Health Sciences, University of Zimbabwe, Harare, Zimbabwe; Department of Harare City Health, Harare, Zimbabwe

**Keywords:** Efavirenz, Central nervous system adverse effects, CYP2B6, HIV, Tuberculosis, Zimbabwe

## Abstract

**Background:**

Efavirenz (EFV) therapeutic response and toxicity are associated with high inter-individual variability attributed to variation in its pharmacokinetics. Plasma concentrations below 1 μg/ml may result in virologic failure and above 4 μg/ml, may result in central nervous system adverse effects. This study used population pharmacokinetics modeling to explore the influence of demographic and pharmacogenetic factors including efavirenz-rifampicin interaction on EFV pharmacokinetics, towards safer dosing of EFV.

**Methods:**

Patients receiving an EFV-based regimen for their antiretroviral therapy and a rifampicin-containing anti-TB regimen were recruited. EFV plasma concentrations were measured by HPLC and genomic DNA genotyped for variants in the *CYP2B6, CYP2A6* and *ABCB1* genes. All patients were evaluated for central nervous system adverse effects characterised as sleep disorders, hallucinations and headaches using the WHO ADR grading system. A pharmacokinetic model was built in a forward and reverse procedure using nonlinear mixed effect modeling in NONMEM VI followed by model-based simulations for optimal doses.

**Results:**

CYP2B6*6 and *18 variant alleles, weight and sex were the most significant covariates explaining 55% of inter-individual variability in EFV clearance. Patients with the CYP2B6*6TT genotype had a 63% decrease in EFV clearance despite their CYP2B6*18 genotypes with females having 22% higher clearance compared to males. There was a 21% increase in clearance for every 10 kg increase in weight. The effect of TB/HIV co-treatment versus HIV treatment only was not statistically significant. No clinically relevant association between *CYP2B6* genotypes and CNS adverse effects was seen, but patients with CNS adverse effects had a 27% lower clearance compared to those without. Model- based simulations indicated that all carriers of CYP2B6*6 TT genotype would be recommended a dose reduction to 200 mg/day, while the majority of extensive metabolisers may be given 400 mg/day and still maintain therapeutic levels.

**Conclusion:**

This study showed that screening for *CYP2B6* functional variants has a high predictability for efavirenz plasma levels and could be used in prescribing optimal and safe EFV doses.

## Background

Treatment success with Efavirenz (EFV) requires maintenance of an optimal plasma concentration to ensure a balance between adverse drug reactions (ADRs) and possible treatment failure. Steady state concentrations below 1 μg/ml in plasma have been reported to be associated with an increased risk for virological failure and drug resistance, while concentrations above 4 μg/ml have been reported to be associated with an increased risk for the development of central nervous system (CNS) adverse effects, hepatic toxicity, and necessity for treatment discontinuation [1,2]. High rates of CNS adverse effects characterized by hallucinations, vivid dreams and insomnia have been reported in more than 50% of the patients who initiate EFV with up to a fifth of all individuals on an EFV based regimen discontinuing the drug and switching therapy primarily due to the unbearable neurotoxicity [3].

The current Zimbabwean guidelines for antiretroviral therapy (ART) recommend first line therapy of EFV at a dosage of 600 mg daily combined with two nucleoside reverse transcriptase inhibitors [4]. Reduced EFV doses of 200 and 400 mg daily have been shown to be effective in patients with good virologic response [5-7]. A randomized, double-blind, placebo-controlled trial (Encore 1) which was conducted in antiretroviral-naive adults showed that a daily dose of 400 mg EFV is non-inferior to the standard 600 mg dose and should be considered for initial ARV treatment [8]. The co-administration of EFV with standard anti-TB therapy that includes rifampicin, a potent drug enzyme inducer, isoniazid, ethambutol and pyrazinamide is recommended for all patients with HIV/AIDS and active TB co-infection [4]. TB is the most frequent life-threatening opportunistic infection among people living with HIV and a leading cause of death [9]. Zimbabwe is ranked among high burden countries for both TB and HIV [10].

The large inter-individual variability in EFV pharmacokinetics (PK) compromises the prediction of associated adverse effects as well as clinical outcomes. The effects of genetics, gender and weight on the variability of EFV PK have been explored previously [11-14]. EFV is primarily metabolized to its main metabolite, 8-hydroxyefavirenz by CYP2B6 [15] and to a lesser extent by CYP3A4 [15] and CYP2A6 [16]. P-glycoprotein, encoded by ABCB1, is the major efflux transporter at the blood brain barrier that limits entry into the CNS for a large number of drugs. There are conflicting reports in literature as to whether EFV is a substrate for Pgp [17,18]. Genetic polymorphisms in these drug metabolizing enzymes and transporter proteins have been associated with variability in EFV PK [14].

Of all the *CYP2B6* variants identified, the CYP2B6*6 haplotype (*516G > T* and *785A > G*) is the most frequent and functionally relevant variant across several populations [19,20], associated with reduced EFV clearance [21,22] and increased CNS adverse effects [23]. A less frequent polymorphism CYP2B6*18 (*983 T > C*), has also been shown to predict plasma EFV exposure [24]. There is limited data available on the additional functional *CYP2B6* polymorphisms that have been suggested to affect EFV PK. Polymorphisms in *CYP2A6*, in particular CYP2A6*9b (1836G > T) and CYP2B6*17 (5065G > A), have been associated with variability in EFV PK [16,25]. There are conflicting reports on the effects of common polymorphisms in the *ABCB1* gene on EFV PK [13,14] with some suggesting a favorable virologic response and CD4-cell recovery in patients carrying the *ABCB1 3435TT* genotype while others failing to replicate this association. There is also contradiction on the effect of EFV-rifampicin interaction on EFV PK with some studies showing an increased metabolism of EFV in the presence of rifampicin [26,27], while others report the opposite [28,29]. Some authors have suggested that isoniazid may play a role in counteracting the inducing effects of rifampicin on EFV metabolism [30]. In contrast, pyrazinamide has been shown not to affect CYP activities thereby not affecting EFV PK [31] and no effects have been reported to date with ethambutol.

Identifying the sources of EFV PK variability may improve therapeutic efficacy while decreasing EFV-induced adverse effects. We recently reported a high incidence of CNS adverse effects associated with carriage of CYP2B6*6TT genotype and male gender in Zimbabwean HIV positive patients on an EFV-based regimen [32]. Due to the large inter-patient variability in EFV concentrations, in combination with a narrow therapeutic index, therapeutic drug monitoring (TDM) has been suggested as a clinically useful monitoring tool during EFV treatment [33]. An alternative and less costly strategy to TDM aims to use patient specific factors (genetic, demographic) to guide dosing so as to achieve optimal drug exposure and effect. Therefore the aim of this study was to investigate the contribution of demographic and pharmacogenetic factors as well as EFV-rifampicin drug interactions on EFV PK using population pharmacokinetic modeling in Zimbabwean patients with HIV/AIDS and TB co-infection. Consequently, the final covariate model was used to simulate optimal EFV doses at various conditions. This study forms a basis for integrating pharmacogenetic testing in routine clinical practice as a step in evaluating drug safety and efficacy.

## Methods

### Study population and sample collection

A total of 95 HIV positive patients receiving an EFV-based ART regimen and 90 HIV/TB co-infected patients receiving an EFV-based ART regimen and a rifampicin-containing anti-TB therapy were recruited and enrolled into the study. Patient recruitment took place at two major hospitals in Zimbabwe; Wilkins and Chitungwiza hospitals. All patients were evaluated for CNS adverse effects in terms of sleep disorders, hallucinations and headaches using a score chart and classified into cases (presence of CNS adverse effects) and controls (no CNS adverse effects). The classification and determination of severity of the CNS side effects was done according to WHO guidelines [34]. Patient demographics were also collected. Blood samples for genotyping and EFV plasma concentration determination were collected at enrollment. The study was approved by the local Joint Research Ethics Committee and Medical Research Council of Zimbabwe. A written informed consent was obtained from each study participant.

### DNA extraction and TaqMan Genotyping

Genomic DNA was isolated from peripheral blood leukocytes using the QIAamp DNA Midi Kit (QIAGEN GmbH.Hilden. Germany). All participants were genotyped for *CYP2B6 G516T* (rs3745274), CYP2B6*18 (rs28399499), CYP2A6*9 (rs8192726), CYP2A6*17 (rs28399454) and *ABCB1 1236C/T* (rs1128503). Allelic discrimination reactions were performed using TaqMan genotyping assays (Applied Biosystems, CA, USA) on the ABI 7500 System (Applied Biosystems, Foster City, CA). The final volume for each reaction was 25 μl, consisting of a 2x TaqMan genotyping master mix, 20 × genotyping assay mix and 10 ng genomic DNA. The PCR profile consisted of an initial step at 50°C for 2 min and 50 cycles with 95°C for 10 minutes and 92°C for 15 sec.

### Efavirenz plasma concentration determination

Plasma EFV concentrations were determined 12–15 hrs post dose by reverse phase HPLC with UV-detection as previously described [35] with minor changes. Briefly, the reverse-phase chromatography with column: C18 (150 x 4.6 mm, 5 μm particle size) and UV/VIS detector (DAD) were used. Stock solutions for the calibration standards (0.5μΜ - 60 μM) were prepared using a mixture of acetonitrile (ACN) and water (dH_2_O) in the ratio 60:40. The quality control (QC) samples were prepared in the same way as the calibration standards to give final concentrations of 2μΜ (Low QC), 30μΜ (Medium QC) and 50μΜ (High QC). Felodipine was used as the internal standard with a retention time of 6.2 minutes. The mobile phase consisted of a mixture of solutions A and B in a 65:35 proportion. Both solutions contained glacial acetic acid, ACN and 25 mM ammonium acetate buffer in proportions 1:900:100 and 1:100:900, respectively. Plasma proteins were precipitated with ACN before centrifuging. Elution was performed at 1 ml/min giving a retention time for EFV of 5.2 min as detected at UV–VIS 1, 247 nm for a total run time of 10mins. Analysis of chromatograms was performed on the Agilent HP1100 HPLC System and data processing was done using the Chemstation Software (Agilent Technologies, CA, USA).

### Statistical analysis of the data

Descriptive analysis of the data was performed using Genstat 8.1 to determine the means and standard deviations for continuous variables and percentages for categorical variables. ANOVA, linear regression and Chi-square/Fisher tests were used to assess the relationship between independent and dependent variables where appropriate. The Shapiro-Wilk test was used to assess for normality and the appropriate data transformation methods used where necessary. All tests perfomed in this section were carried at 95% confidence level and p < 0.05.

### Population pharmacokinetic modeling

Pharmacokinetic data was analyzed using population mixed effects non-linear regression modeling in NONMEM VI. The estimation of typical population PK parameters, along with their random inter-individual and inter-occasional (IO) variability was performed using first-order conditional estimation method with interaction (FOCE INTER) [36]. The base model was built with all covariates and tested for significant relationships between parameters and covariates. The baseline EFV PK model parameters were adopted from a study in Zimbabwean patients by Nyakutira *et al.* [37]. Clearance (CL/F) was the only parameter that was estimated while the first-order absorption rate constant (ka) and volume of distribution in plasma (Vd) were fixed. A stepwise regression method was used and the statistical significance set at 5% (change in objective function value (ΔOFV) > 3.84,1.degrees of freedom [d.f] ) and 1% significance level (ΔOFV > 6.63, 1.d.f) for the forward and backward inclusion of covariates respectively [38]. Clinical significance was set at 20%.The effect of continuous covariates was parameterized centred on the median value using the following equation:

$$ PAR={\uptheta}_P\times \left(1 + {\uptheta}_{cov}\times \left(COV-CO{V}_{med}\right)\right) $$

where θ_*P*_ is the parameter (*PAR*) estimate in a typical individual, *COV*_*med*_ is a median covariate value while θ_*cov*_ is the fractional change in *PAR* with each unit change in the covariate (*COV*).

For categorical covariates, such as genotype and sex, the covariate model was expressed as a fractional change (θ_*cov*_) from the estimate for a typical value (θ_*P*_) due to the covariate (COV) using the following equation:

$$ PAR={\uptheta}_P\times \left(1+{\uptheta}_{cov}\times (COV)\right) $$

#### Monte-carlo simulations

To propose dose adjustment the PK data was simulated in NONMEM VI on 1000 individuals using the final model parameters mimicking EFV drug concentration on demographic and genetic data from the 185 individuals at different doses: 200, 300, 400, 500, 600, 700 and 800 mg oral per day. A dose was selected that minimized the proportion of patients outside the 1 - 4 μg/ml therapeutic range.

## Results

### Descriptive statistics

A total of 185 patients; 60 males and 125 females; were recruited into the study and used for data analysis. The mean weight and height were significantly higher for males than females (61.5 kg vs 57.9 kg; p=0.0372 and 1.72 m vs 1.61 m; p<0.001, respectively). There was no statistically significant difference in occurrence of CNS toxicity and mean EFV concentration between males and females. The summary characteristics of the study participants are presented in Table 1.

**Table 1:** **Demographic characteristics of the study population**

	**N=185**		
**Characteristics**	**Males, n=60**	**Females n=125**	**P value**
Age (years)	40.16667 (9.141)	38.336 (8.065)	0.1683
Weight (kg)	61.51667 ( 10.058)	57.92 (11.291)	0.0372
Height (m)	1.718644 (0.089)	1.607258 (0 .084)	<0.001
Duration on EFV (months)	6.941667 (9.967)	10.30456 (12.272)	0.0661
CNS Toxicity,			
Yes	26 [32.91]	53 [67.09]	
No	34 [32.08]	72 [67.92]	0.904
Log EFV concentration	1.382 (1.274)	1.632 ( 1.069)	0.1638
Genetic polymorphisms			
CYP2B6*6 ,			
GG	17 [29.82]	40 [70.18]	
GT	29 [34.52]	55 [65.48]	
TT	13 [33.33]	26 [66.67]	0.841
CYP2B6*18,			
TT	43 [32.58]	89 [67.42]	
TC	14 [29.79]	33 [70.21]	
CC	3 [50.00]	3 [50.00]	0.608
CYP2A6*9,			
GG	33 [30.00]	77 [70.00]	
TT	8 [33.33]	16 [66.67]	0.748
CYP2A6*17,			
GG	38 [31.67]	82 [68.33]	
GA	3 [20.00]	12 [80.00]	
AA	1 [100]	0 [0]	0.212
*ABCB1236 C/T*			
CC	37 [32.17]	78 [67.83]	
CT	5 [31.25]	11 [68.75]	
TT	0 [0]	6 [100]	0.316

The distribution of study variable outcomes grouped by sex and presented as mean and standard deviation (SD) for continuous variables and total number and [%] for categorical variables.

**Figure 1:**
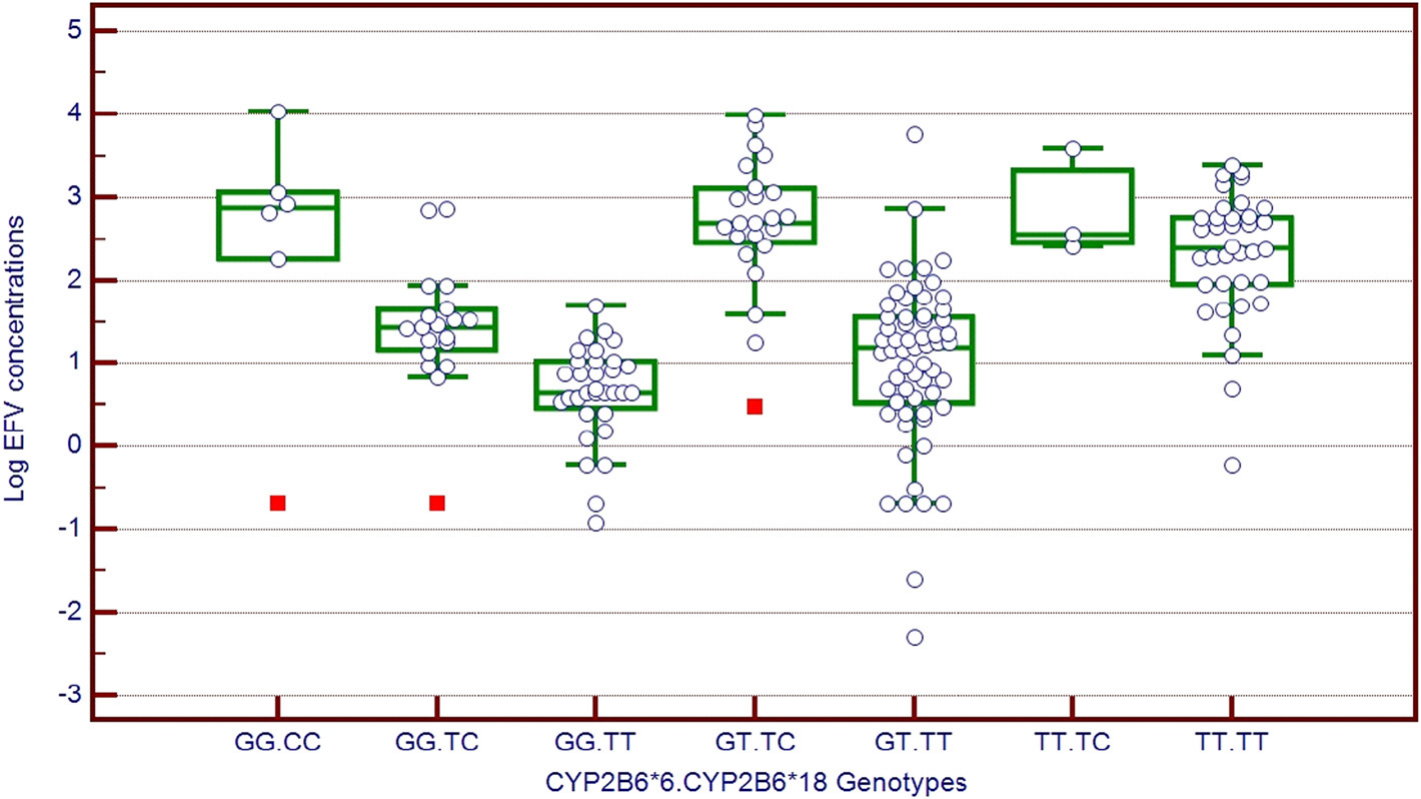
**Log EFV concentration among the** CYP2B6*6 and *18 composite genotypes.

All identified SNPs were tested for deviations from Hardy-Weinberg Equilibrium. Analysis of the log transformed EFV concentration and categorical explanatory variables revealed significantly higher mean log EFV concentration for patients carrying the homozygous mutant genotypes for CYP2B6*6, CYP2B6*18 and CYP2A6*9 compared to the other genotypes (Table 2). Figure 1 shows the mean log EFV concentration among the combined genotypes of CYP2B6*6 and *18. Patients carrying the homozygous mutant genotypes and at least two of the mutant alleles showed a fourfold higher plasma EFV concentration than those carrying the homozygous wild type genotypes. In addition most of the patients carrying the homozygous wild type genotypes had EFV levels that were in the therapeutic range log_10_ (0 – 0.5 μg/ml), corresponding to 1 - 4 μg/ml.

**Table 2:** **Association between log transformed EFV concentration and categorical explanatory variables.**

**Variable**	**Mean Log EFV concentration**	**p-value**	**CV**
Gender		0.195	74%
Male	1.68		
Female	1.46		
CYP 2B6*6		0.008*	68%
GG	1.16		
GT	1.55		
TT	2.35		
CYP 2B6*18		<0.001***	69%
TT	1.35		
TC	2.22		
CC	3.01		
*ABCB1 1236C/T*		0.841	74%
CC	1.60		
CT	1.56		
TT	1.30		
CYP 2A6*9		0.004**	72%
GG	1.47		
AA	2.21		
CYP 2A6*17		0.694	74%
AA	1.92		
AG	1.45		
GG	1.62		
CNS Toxicity		0.122	73%
Yes	1.72		
No	1.43		
Regimen		0.619	73%
TDF/3TC/EFV	1.434		
AZT/3TC/EFV	1.510		
D4T/3TC/EFV	1.655		
ART Only	1.452	0.334	79%
ART + anti-TB Therapy	1.618		

“*”p < 0.05, “**”p < 0.01, “***”p < 0.001; CV= coefficient of variation.

However, the mean log EFV concentration for patients who experienced CNS adverse effects was comparable to that of patients without. Analysis of the EFV concentration against the continuous variables revealed no significant association with age and height but there was a significant decrease in concentration for each unit increase in the weight (p<0.001). The association between log transformed EFV concentration and categorical explanatory variables is summarized in Table 2.

### Pharmacokinetic parameter estimation

A one compartmental PK model was used to estimate the impact of multi-covariates to the fixed parameter EFV CL/F. The ka and V/d were fixed in the model based on literature results. Covariates that resulted in statistically significant decreases in the baseline PK model were polymorphisms CYP2B6*6, CYP2B6*18, body weight and sex resulting in ∆OFV from 1.098 to 0.494; (p>0.001), explaining up to 55% of between subject variability in clearance. The parameter estimates for the final PK model for a daily dose of 600 mg EFV are shown in Table 3.

**Table 3:** **Parameter estimates for the final pharmacokinetic model for daily 600 mg EFV**

**Parameter**	**Estimate (% RSE)** ^**a**^	^**b**^ **IIV (%RSE)**
CL/F_TT_ (L/hr)	7.01 (10)	70.3 (7)
CL/F_TC_ (L/hr)	2.26 (12)	70.3 (7)
C/FL_CC_ (L/hr)	0.539 (24)	70.3 (7)
V/F (L/hr)	150 FIX	
ka (hr ^−1^)	0.18 FIX	
PROP_ERR	0.12	
*Effect on CL/F*_*TT*_ *, CL/F*_*TC*_ *and CL/F*_*CC*_		
CYP2B6_GG_ (%)	+93.1 (24%)	
CYP2B6_TT_ (%)	−63.4 (9)	
CYP2B6_GT_ (%)	0	
10 kg increase body weight (%)	+21.1 (21)	
Females (%)	+22.2 (67)	

^a^RSE, relative standard error (how precise the model is estimating the IIV). ^b^IIV, inter-individual variability reported as percent coefficient of variation. CL/F, oral clearance; CYP2B6*18 TT, extensive metabolizer; *TC*, Intermediate metabolizer; *CC*, poor metabolizer; V/F, volume of distribution; ka, first-order absorption rate constant; PROP_ERR, proportional error; *CYP2B6 GG*, extensive metabolizer; *GT*, Intermediate metabolizer; *TT*, poor metabolizer.

**Figure 2:**
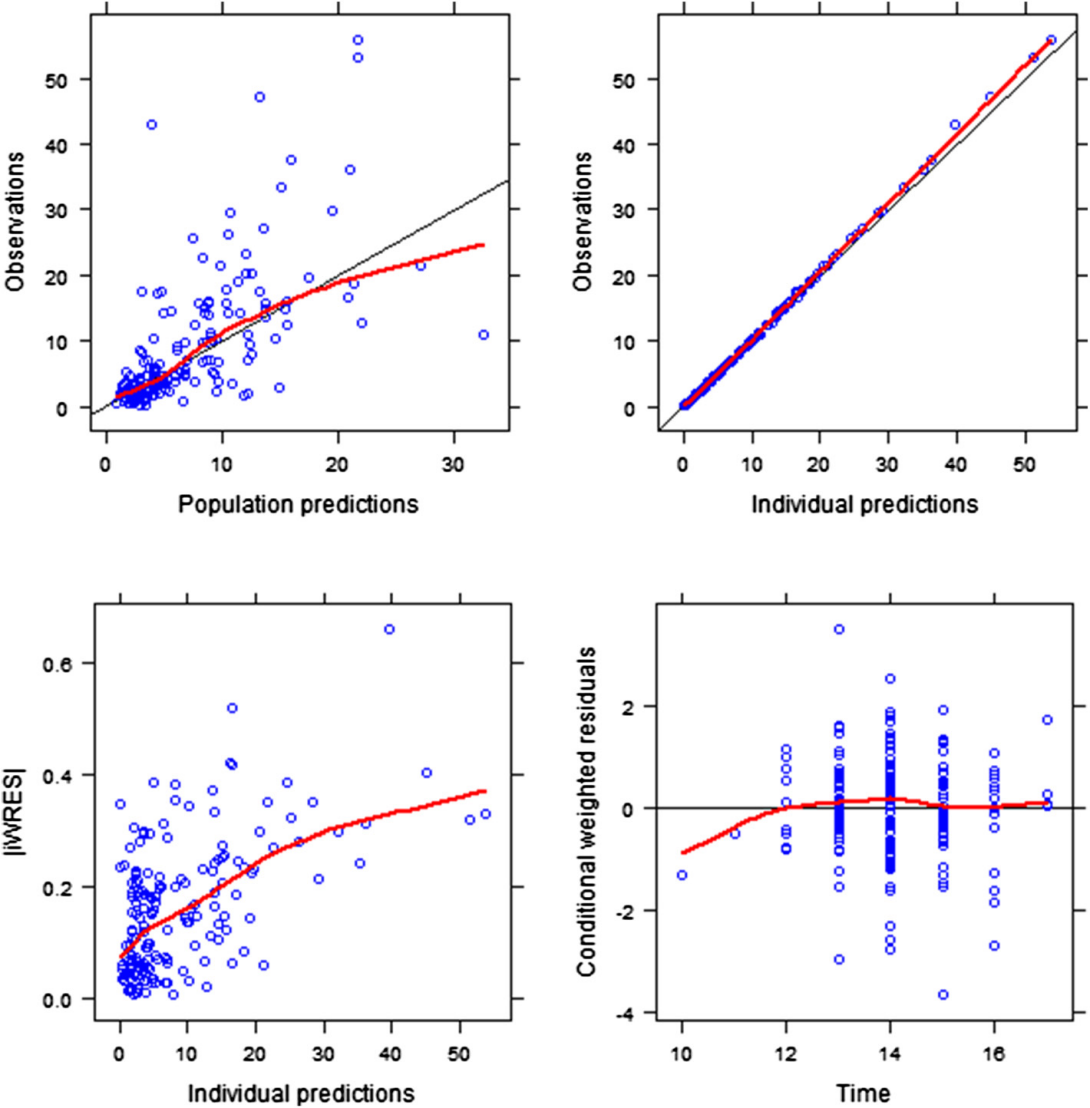
**Basic goodness of fit plots for the final EFV PK model.** The observations are plotted versus the population predictions. Upper right panel: The observations are plotted against the individual predictions. Lower left panel: The individually weighted residuals are plotted versus time after dose. Lower right panel: The absolute values of the individually weighted residuals are seen versus the individual predictions. The predictions match the observations and the residuals are distributed evenly around the reference line over time and do not give a pronounced slope over the predicted concentration range.

The most significant covariate was CYP2B6*18 with ∆OFV from 1.098 to 0.901 accounting for up to 18% variance in EFV clearance. The contribution of the covariate towards explaining between subject variability is shown in Table 4. As a result quantification of EFV oral clearance was fixed on CYP2B6*18. For the extensive metabolisers, CL/F was 7.01 L/h which significantly decreased to 2.26 L/h and 0.539 L/h in intermediate and poor metabolisers, respectively. Carriers of the CYP2B6*6 wild type had a 93% higher CL/F (CV=24%) while the poor metabolisers, CYP2B6*6 TT had 63% lower CL/F (CV=9%). For every 10 kg increase in weight the CL/F increased by 21% (CV=21%). Females showed a 22% higher CL/F (CV= 67%) compared to males. The final model adequately explained the observed data as shown by the basic goodness-of-fit plots for the model evaluation shown in Figure 2.

**Table 4:** **Table showing contribution of each covariate on improving the model fit and percentage of inter-individual variability on EFV clearance accounted for**

**Covariate**	**Points decrease in OFV** ^**a**^	**DF** ^**b**^	**p-value**	**IIV explained (%)** ^**c**^
CYP2B6*18	67.07	2	<0.0001	18.0
CYP2B6*6	36.03	2	<0.0001	16.4
Body weight	19.37	1	<0.0001	10.7
Sex	10.41	1	0.0013	9.9
Age	0.29000	1	0.590220	0.01
EFV-RIF interaction^d^	1.93200	1	0.164540	1.1
CNS effect	1.01100	1	0.314660	0.14
CYP2A6*9	3.83600	1	0.050163	2.7
CYP 2A6*17	1.44	2	0.230140	1.8

^a^Change in NONMEM objective function value. ^b^Degrees of freedom. ^c^Inter- individual variability. ^d^Interaction between efavirenz and rifampicin.

#### Monte-Carlo dose simulations

A reduction in EFV dose from 600 mg/dy to 400 mg/dy for the CYP2B6 extensive metabolisers would still result in an effective EFV exposure (1 - 4 μg/ml) for most patients. CYP2B6*6 GT carriers would require doses between 200 – 400 mg/dy depending on the CYP2B6*18 genotype, their gender and weight. All CYP2B6*6 TT carriers irrespective of their CYP2B6*18 genotype, weight and gender would require a reduced daily dose of 200 mg. The proposed optimal doses obtained from the simulation studies are summarized in Table 5.

**Table 5:** **Proposed optimal doses given** ***CYP2B6*** **genotypes, weight and gender**

**Variable**		**Females**		**Males**	
		<58 kg	>58 kg	<58 kg	>58 kg
CYP2B6*18	CYP2B6*6	1 -4 μg/ml	1 -4 μg/ml	1 -4 μg/ml	1 -4 μg/ml
TT	GG	400	400	400	400
TT	GT	200	200	200	200
TT	TT	200	200	200	200
TC	GG	400	400	400	400
TC	GT	400	200	200	200
TC	TT	200	200	200	200
CC	GG	400	600	600	600
CC	GT	200	300	300	300
CC	TT	200	200	200	200

## Discussion

In the present study, the use of mixed effects modelling enabled the assessment of potential demographic and pharmacogenetic factors on EFV Cl. Consequently the final PK model was used to simulate therapeutic EFV doses associated with reduced occurrence of CNS side effects. The CYP2B6*6 and *18 variant alleles have been reported to show significant correlation with high EFV concentrations, with the CYP2B6*6 allele as a main risk factor for plasma EFV levels above 4 μg/ml [24,37]. Our results showed that the combined CYP2B6 SNPs had a clinically significant additive effect on reducing EFV CL and were associated with an almost four-fold higher EFV concentration, a finding in agreement with reports by Wyen *et al.* [39] and Maimbo *et al.* [24]. Similar observations were made in Caucasians, Africans and Asians [11,23,40,41].

An earlier report by Nemaura *et al.* showed that weight and gender combined with CYP2B6*6 polymorphisms can explain 22% of variability in EFV PK [42]. This was replicated in our study and we further showed that addition of more clinically significant factors can increase the percentage of variability being explained. Our final model was able to explain up to 55% of IIV. Although previous reports have indicated that females have a lower EFV CL compared to males [37,42], our results showed that females had a 22% higher clearance of EFV compared to the males. A study done in Hispanic women showed that they had increased CYP2B6 metabolic capacity due to some SNPs in the regulatory regions of the gene resulting in more CYP2B6 mRNA [43]. This may also explain our finding. There is need therefore, to determine these SNPs before a conclusion can be made regarding gender differences in the expression and activity of CYP2B6.

Our results did not identify polymorphisms of *CYP2A6* and *ABCB1* as significant covariates in the final model. There are currently conflicting reports in literature over the effect of EFV interaction with rifampicin. Both EFV and rifampicin are inducers of *CYP2B6* and *CYP3A4*, which can lead to drug–drug interactions and decreased exposure of the drug. Some reports show increased metabolism of EFV in the presence of rifampicin consequently lowering EFV exposure [44]. Some authors suggest that the interaction may be modified by other anti-tubercular agents such as isoniazid which has been shown to inhibit many CYP P450 enzymes including *CYP3A* thereby counter balancing the inducing effect of rifampicin [30]. Our present results revealed no statistically significant difference in EFV concentration for patients on HIV treatment only and for those on HIV/TB co-treatment containing rifampicin.

Another observation from our study is that an increase in body weight after the time of initial measurement results in a decrease in EFV concentration, which agrees with a study in Thais which showed that body weight was an independent predictive factor for plasma EFV concentration [45,46], although some previous studies have not demonstrated this effect [29,47]. Since patients’ body weights may increase over time while on treatment, a weight-based cutoff for EFV dosing is a practical therapeutic approach. To date, a body weight cutoff of 60 kg for the standard EFV dosing is recommended.

With regards the occurrence of CNS adverse effects, our analysis did not show a clinically significant association between the CYP2B6*6 and *18 genotypes and occurrence of CNS adverse effects although patients with CNS side effects had a 27% lower EFV Cl compared to those without. This result shows that other non-genetic factors play a role in development of these side effects. The occurrence and progression of symptoms for CNS side effects after administration of EFV also pose a challenge due to the wide range in time of symptoms onset and persistence. A report by Rodriguez-Novoa *et al.* showed that carriers of the *CYP2B6 516 T* allele have greater plasma EFV levels during the first 24 weeks of ART, and they experienced frequent CNS-related side effects during the first week of treatment [41]. Other studies show that symptoms may emerge after weeks of treatment and persist for several months [48]. In other reports, patients develop tolerance of side effects despite continued high EFV concentrations. A blinded, placebo-controlled study by Clifford et al., showed that with optimal use of EFV, stable or improved neurological performance is generally achieved for patients who remain on treatment over more than 3 years [49]. Similar patterns were observed in our study where patients developed symptoms from four weeks after EFV initiation and in some patients symptoms persisted for up to 72 months. Given this challenge, it is difficult to optimize a sampling window period for CNS side effects which may result in failure to associate their occurrence with the CYP2B6*6 and *18 genotypes. It is therefore crucial to replicate findings of the phenotype-genotype association study in a well controlled clinical study with sensitive screening tests for detection of the CNS side effects.

In order to minimize the occurrence of CNS side effects, a gradual reduction in the dose of efavirenz from 600 to 400 or 200 mg/day for intermediate and poor metaboliser patient groups, respectively, have been proposed [50]. Earlier studies have recommended increasing the EFV dosage to 800 mg/day in patients receiving EFV and rifampicin concomitantly [51] but later studies have demonstrated the efficacy of the recommended 600 mg/day. Recently some studies have suggested that the dosage be increased to 800 mg/day in patients weighing >50 kg [52]. Our simulation results show that reductions in EFV dose from 600 mg/day to 400 mg/day would still maintain the therapeutic range of the drug for most of the extensive metaboliser patient groups. This is in agreement with an earlier modeling study on the effectiveness of 400-mg efavirenz vs a 600 mg dose [8]. Daily doses of between 200–400 mg may be recommended for the poor metaboliser patient group but there is still need to closely monitor these patients to avoid sub-therapeutic concentrations leading to virologic failure.

## Conclusion

CYP2B6*6 and *18 polymorphisms, gender and weight are predictors of EFV PK variability, and can explain up to 55% of the inter-individual variability. Our findings form a basis to start addressing EFV efficacy and safety in our population through carefully planned clinical trials to validate these predictive factors. There is need for a thorough investigation on the EFV-rifampicin interaction by also including the polymorphisms in the *N-acetyltransferase* 2 (NAT2) and their implications on isoniazid metabolism. Perhaps inclusion of more factors, genetic and non-genetic may help to explain the remaining 45% of inter individual variability. Close follow up and regular TDM of plasma EFV concentrations during early therapy is recommended, especially in patients with the underlying risk factors for early diagnosis and management of efavirenz-based ART induced CNS adverse effects.
